# Acute Neurological Deterioration in a Child with Shunt-Dependent Post-Hemorrhagic Hydrocephalus: A Case Report

**DOI:** 10.3390/children13091138

**Published:** 2026-08-25

**Authors:** Ahmad Kharoufeh, Riyam Aljorani, Mohammed Dalbah, Leen Gafar, Haidy Alzaghal, Malak Abedi, Mohmed Doukarli, Subhranshu Sekhar Kar, Rajani Dube, Mohamed Anas Patni, Hussein Eleimy

**Affiliations:** 1 RAK College of Medical Sciences, Ras Al Khaimah Medical and Health Sciences University, Ras Al Khaimah P.O. Box 11172, United Arab Emirates; riyam.24901121@rakmhsu.ac.ae (R.A.); leen.24901050@rakmhsu.ac.ae (L.G.); haidy.24901036@rakmhsu.ac.ae (H.A.); malaek.21901100@rakmhsu.ac.ae (M.A.); mohmed2290184@rakmhsu.ac.ae (M.D.); 2 Department of Pediatrics, RAK College of Medical Sciences, Ras Al Khaimah Medical and Health Sciences University, Ras Al Khaimah P.O. Box 11172, United Arab Emirates; mohamed.dalbah@ehs.gov.ae; 3 Department of Pediatrics and Neonatology, RAK College of Medical Sciences, Ras Al Khaimah Medical and Health Sciences University, Ras Al Khaimah P.O. Box 11172, United Arab Emirates; subhranshu.kar@rakmhsu.ac.ae; 4 Department of Obstetrics and Gynecology, RAK College of Medical Sciences, Ras Al Khaimah Medical and Health Sciences University, Ras Al Khaimah P.O. Box 11172, United Arab Emirates; rajani.dube@rakmhsu.ac.ae; 5 Department of Community Medicine, RAK College of Medical Sciences, Ras Al Khaimah Medical and Health Sciences University, Ras Al Khaimah P.O. Box 11172, United Arab Emirates; 6 Department of Pediatrics, Fujairah Hospital, Fujairah P.O. Box 11172, United Arab Emirates; hussien.eleimy@ehs.gov.ae

**Keywords:** post-hemorrhagic hydrocephalus, ventriculoperitoneal shunt, intraventricular hemorrhage, pediatric hydrocephalus, VP shunt complications, shunt malfunction, suspected VP shunt infection, status epilepticus, secondary adrenal insufficiency

## Abstract

**Highlights:**

**What are the main findings?**

- A shunt-dependent child with post-hemorrhagic hydrocephalus developed acute neurological deterioration in the setting of several competing neurological and systemic processes.
- Persistently negative microbiological cultures, initially unchanged ventricular size, status epilepticus, and evolving bilateral extra-axial collections created substantial diagnostic uncertainty. VP shunt-related infection or malfunction remained important but unconfirmed diagnostic considerations, and no single etiology was definitively established.

**What are the implications of the main findings?**

- Acute neurological deterioration in a shunt-dependent child should be evaluated using a broad differential diagnosis rather than attributed to shunt-related pathology on the basis of individual clinical, microbiological, or radiological findings.
- Serial neurological assessment, repeat neuroimaging, appropriate laboratory and microbiological investigation, and multidisciplinary evaluation are important when the initial assessment does not establish a definitive cause.

**Abstract:**

Post-hemorrhagic hydrocephalus (PHH) is a serious neurological sequela of severe intraventricular hemorrhage (IVH) in premature infants and remains one of the leading indications for ventriculoperitoneal (VP) shunt placement. Evaluating possible VP shunt-related complications can be challenging because clinical manifestations are often nonspecific, neuroimaging may initially appear unchanged, and microbiological cultures may remain negative. We report the case of a 19-month-old male born at 28 weeks’ gestation who developed Grade IV germinal matrix/intraventricular hemorrhage with bilateral intraparenchymal extension, early periventricular cystic leukomalacia, and post-hemorrhagic communicating hydrocephalus requiring multiple cerebrospinal fluid diversion procedures culminating in long-term VP shunt dependence. His medical history was notable for recurrent neonatal meningitis, secondary epilepsy with previous episodes of status epilepticus, secondary adrenal insufficiency, and severe global developmental delay. He presented with fever, recurrent coffee-ground vomiting, abdominal distension, progressive lethargy, reduced responsiveness, and localized erythematous swelling over the cranial VP shunt reservoir, raising concern for possible shunt-related pathology. During hospitalization, he deteriorated with status epilepticus and respiratory failure, with clinical concern for increased intracranial pressure, requiring admission to the Pediatric Intensive Care Unit (PICU). Laboratory investigations demonstrated leukocytosis, elevated C-reactive protein, cerebrospinal fluid pleocytosis, markedly elevated CSF protein, and CSF glucose of 2.0 mmol/L, for which a paired serum glucose value was unavailable, while repeated blood, urine, wound, and CSF cultures remained negative. Initial computed tomography (CT) demonstrated no significant interval change in the chronic hydrocephalus despite progressive neurological deterioration; however, serial neuroimaging later revealed progressive bilateral extra-axial fluid collections with radiological features suggestive of an evolving subacute subdural hemorrhage. The patient was managed with empirical broad-spectrum intravenous antibiotics, aggressive seizure control, stress-dose corticosteroids, respiratory support, and continuous multidisciplinary monitoring. His neurological and respiratory status subsequently improved, and he returned to his pre-admission neurological baseline before discharge with planned further evaluation at a tertiary pediatric neurosurgical center. This case highlights the diagnostic uncertainty surrounding acute neurological deterioration in a child with shunt-dependent PHH. VP shunt-related infection or malfunction remained important but unconfirmed diagnostic considerations, alongside competing or potentially overlapping contributors including status epilepticus, evolving extra-axial collections, respiratory infection, and endocrine or metabolic decompensation. No single etiology was definitively established. The case emphasizes the importance of serial neurological assessment, consideration of alternative diagnoses, repeat neuroimaging, and multidisciplinary evaluation when initial investigations do not establish the cause of deterioration.

## 1. Introduction

Post-hemorrhagic hydrocephalus (PHH) is one of the most serious neurological complications of severe intraventricular hemorrhage (IVH) in premature infants and remains a leading indication for ventriculoperitoneal (VP) shunt placement [1,2,3,4,5,6,7]. Although VP shunts provide effective long-term cerebrospinal fluid (CSF) diversion, shunt-related complications, including infection, mechanical malfunction, and obstruction, remain major causes of morbidity and emergency hospital admission in children with shunt-dependent hydrocephalus [5,7,8,9,10].

Diagnosing VP shunt complications can be particularly challenging because presenting symptoms such as fever, vomiting, lethargy, irritability, seizures, and altered consciousness are nonspecific and may overlap with underlying neurological impairment or other acute illnesses [9,10,11,12,13]. Microbiological cultures may remain negative in shunt-related infection, particularly following prior antimicrobial therapy or in association with biofilm-forming organisms [12,13,14,15]. Similarly, ventricular enlargement may not accompany shunt malfunction in children with chronic hydrocephalus [9,10,11]. Consequently, clinical findings, microbiological investigations, and neuroimaging must be interpreted together and within a broader clinical context.

Diagnostic uncertainty is particularly pronounced in children with complex neurological and systemic comorbidities, in whom seizure-related, infectious, respiratory, endocrine, metabolic, and structural intracranial processes may produce overlapping manifestations. Distinguishing among these possibilities is clinically important because management may differ substantially according to the underlying cause. Serial neurological assessment, laboratory investigation, repeat neuroimaging, and multidisciplinary evaluation may therefore be necessary when the initial assessment does not establish a definitive diagnosis.

We report a diagnostically challenging case of acute neurological deterioration in a 19-month-old child with shunt-dependent PHH and multiple neurological and systemic comorbidities. VP shunt-related infection or malfunction remained important but unconfirmed considerations within a broader differential diagnosis. The purpose of this report is to illustrate the diagnostic challenges encountered when acute neurological deterioration in a shunt-dependent child cannot be attributed to a single established cause and to emphasize the importance of integrating serial clinical, laboratory, and radiological findings within a broad differential diagnosis.

## 2. Case Presentation

### 2.1. Patient History and Baseline Status

A 19-month-old male born at 28 weeks’ gestation presented with acute neurological deterioration against a background of complex neurosurgical and neurodevelopmental disease. His neonatal course was complicated by Grade IV germinal matrix/intraventricular hemorrhage (IVH) with bilateral intraparenchymal extension, which progressed to moderate-to-marked communicating post-hemorrhagic hydrocephalus (PHH), necessitating permanent cerebrospinal fluid (CSF) diversion with a ventriculoperitoneal (VP) shunt during infancy. Early brain MRI at 4 weeks of age demonstrated periventricular cystic leukomalacia, marked thinning of the corpus callosum, and ballooning of the fourth ventricle, consistent with severe hemorrhagic brain injury and evolving hydrocephalus.

His medical history was additionally notable for recurrent neonatal pyogenic meningitis involving multiple organisms, secondary epilepsy with previous episodes of status epilepticus, secondary adrenal insufficiency requiring long-term corticosteroid replacement, severe global developmental delay, and spastic quadriplegia. His long-term medications included carbamazepine, clonazepam, baclofen, clonidine, hydrocortisone, and fludrocortisone.

### 2.2. Surgical and Interventional Chronology

**Figure 1:**
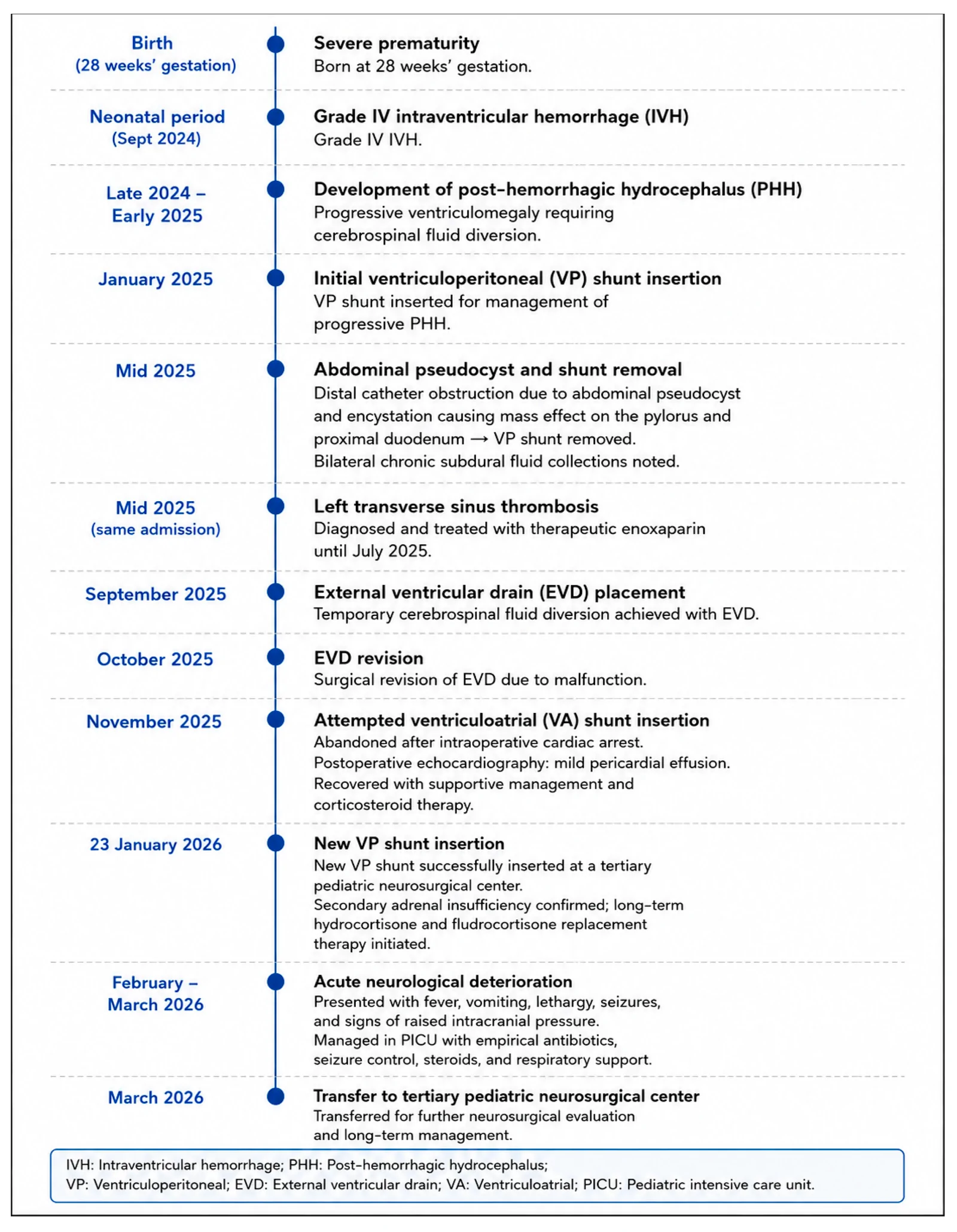
Clinical and neurosurgical timeline illustrating the patient’s course from extreme prematurity and Grade IV intraventricular hemorrhage through post-hemorrhagic hydrocephalus, sequential cerebrospinal fluid diversion procedures, acute neurological deterioration, and subsequent referral for further evaluation at a tertiary pediatric neurosurgical center.

The patient underwent multiple CSF diversion procedures for shunt-dependent post-hemorrhagic hydrocephalus, including VP shunt insertion and subsequent revisions for shunt-related complications. Temporary external ventricular drainage was also required, and an attempted ventriculoatrial shunt insertion in November 2025 was abandoned following intraoperative cardiac arrest. Most recently, a new VP shunt was inserted at a tertiary pediatric neurosurgical center on 23 January 2026. The major neurosurgical interventions and clinically significant events are summarized chronologically in Figure 1.

### 2.3. Pre-Admission Functional Status

Before the current illness, the patient had severe neurological disability but remained clinically stable relative to his baseline. He was awake for prolonged periods, opened his eyes spontaneously, and demonstrated limited interaction with familiar caregivers despite being non-verbal. He remained dependent on nasogastric feeding and required full assistance with all activities of daily living.

Although developmental progress was profoundly delayed, seizure control had remained relatively stable on maintenance antiepileptic therapy, and adrenal insufficiency was adequately managed with physiological corticosteroid replacement.

### 2.4. Acute Clinical Presentation

The patient presented to the emergency department in February 2026 after two days of progressive clinical deterioration.

According to his parents, he initially developed persistent fever associated with recurrent coffee-ground vomiting and progressively worsening abdominal distension. Feeding intolerance rapidly progressed, and he became increasingly irritable before developing marked lethargy, reduced environmental interaction, and decreased responsiveness.

The family also reported a productive cough accompanied by increasing oxygen requirements during the preceding 24 h. Given his history of hydrocephalus and VP shunt dependence, possible shunt-related pathology was considered alongside other neurological, infectious, respiratory, endocrine, and metabolic causes of deterioration.

Shortly after presentation, his neurological condition deteriorated further. Generalized tonic–clonic seizures developed and rapidly progressed to status epilepticus, accompanied by persistent fever, severe sinus tachycardia, and worsening consciousness. Electroencephalography (EEG) was not performed during the reported acute episode. The combination of systemic inflammatory features, neurological decline, and previous neurosurgical history prompted immediate multidisciplinary evaluation involving pediatric intensive care, neurology, neurosurgery, infectious diseases, endocrinology, and pediatric surgery.

At presentation, the principal differential diagnoses included VP shunt infection or malfunction, ventriculitis, meningitis, raised intracranial pressure, adrenal crisis, aspiration pneumonia, and seizure-related metabolic decompensation.

### 2.5. Initial Clinical Examination

On arrival, the patient appeared acutely ill with depressed consciousness and significant neurological compromise.

Vital signs demonstrated persistent pyrexia with marked sinus tachycardia. Neurological examination revealed reduced responsiveness with progressive deterioration in Glasgow Coma Scale (GCS), although no new focal neurological deficits were identified initially. During subsequent deterioration, intermittent up-rolling of the eyes, generalized tonic–clonic movements, and prolonged seizure activity consistent with status epilepticus were observed.

A notable examination finding was the presence of a firm, erythematous, tender swelling directly over the right-sided cranial VP shunt reservoir. Palpation produced marked irritability and discomfort. Although this finding raised concern for a localized process involving the shunt region, it was not specific for shunt infection or mechanical malfunction and was interpreted in conjunction with the other clinical and investigative findings.

Abdominal examination demonstrated mild-to-moderate distension but remained soft without guarding, rebound tenderness, or other signs of peritonitis despite the patient’s previous history of abdominal pseudocyst formation.

Respiratory examination identified increasing oxygen requirements together with productive cough, findings that later correlated with radiographic evidence of aspiration pneumonia following recurrent vomiting and prolonged seizures.

Overall, the combination of fever, vomiting, altered consciousness, localized shunt-region swelling, respiratory symptoms, and evolving status epilepticus prompted urgent evaluation for multiple potential causes of neurological deterioration, including but not limited to VP shunt-related infection or malfunction.

## 3. Investigations

### Laboratory Findings

Initial laboratory investigations demonstrated evidence of a systemic inflammatory response, although no single investigation definitively established the underlying diagnosis.

Hematological analysis revealed leukocytosis with neutrophilia, accompanied by elevated C-reactive protein (CRP) levels, supporting the presence of an inflammatory process. Liver function tests showed transient elevations of aspartate aminotransferase (AST) and alanine aminotransferase (ALT), findings considered consistent with acute systemic illness rather than primary hepatic pathology.

As part of the evaluation for possible VP shunt-related infection, cerebrospinal fluid (CSF) was obtained through sterile aspiration of the shunt (Ommaya) reservoir. Lumbar puncture was deferred following neurosurgical consultation because of clinical concern for increased intracranial pressure and the potential risk of cerebral herniation. No opening-pressure measurement was obtained. Fundoscopic examination was attempted; however, the fundus could not be adequately visualized because of reactive pupils. Therefore, the presence or absence of papilledema could not be determined. The principal laboratory and CSF findings during the acute presentation are summarized in Table 1.

CSF analysis demonstrated markedly elevated protein (>2500 mg/L), CSF glucose of 2.0 mmol/L, and a white blood cell count of 49 cells/mm^3^ with 99% lymphocytes. These abnormalities were nonspecific and did not independently establish an acute shunt-related infection. Interpretation was further complicated by the patient’s history of recurrent neonatal meningitis and chronically elevated baseline CSF protein levels. Consequently, the markedly elevated CSF protein could not be confidently attributed to an acute infectious process.

All microbiological investigations remained negative throughout hospitalization. Repeated blood, urine, wound, and shunt-derived CSF cultures demonstrated no microbial growth, and respiratory viral screening was negative. VP shunt-related infection or ventriculitis remained clinically suspected and was treated empirically; however, infection was not microbiologically confirmed. No documented manifestations of sepsis were identified during the admission.

The combination of inflammatory laboratory abnormalities and negative cultures contributed to the diagnostic uncertainty and required interpretation alongside the patient’s neurological course, neuroimaging findings, and competing infectious, neurological, endocrine, and metabolic considerations [12,14,15].

## 4. Imaging

**Figure 2:**
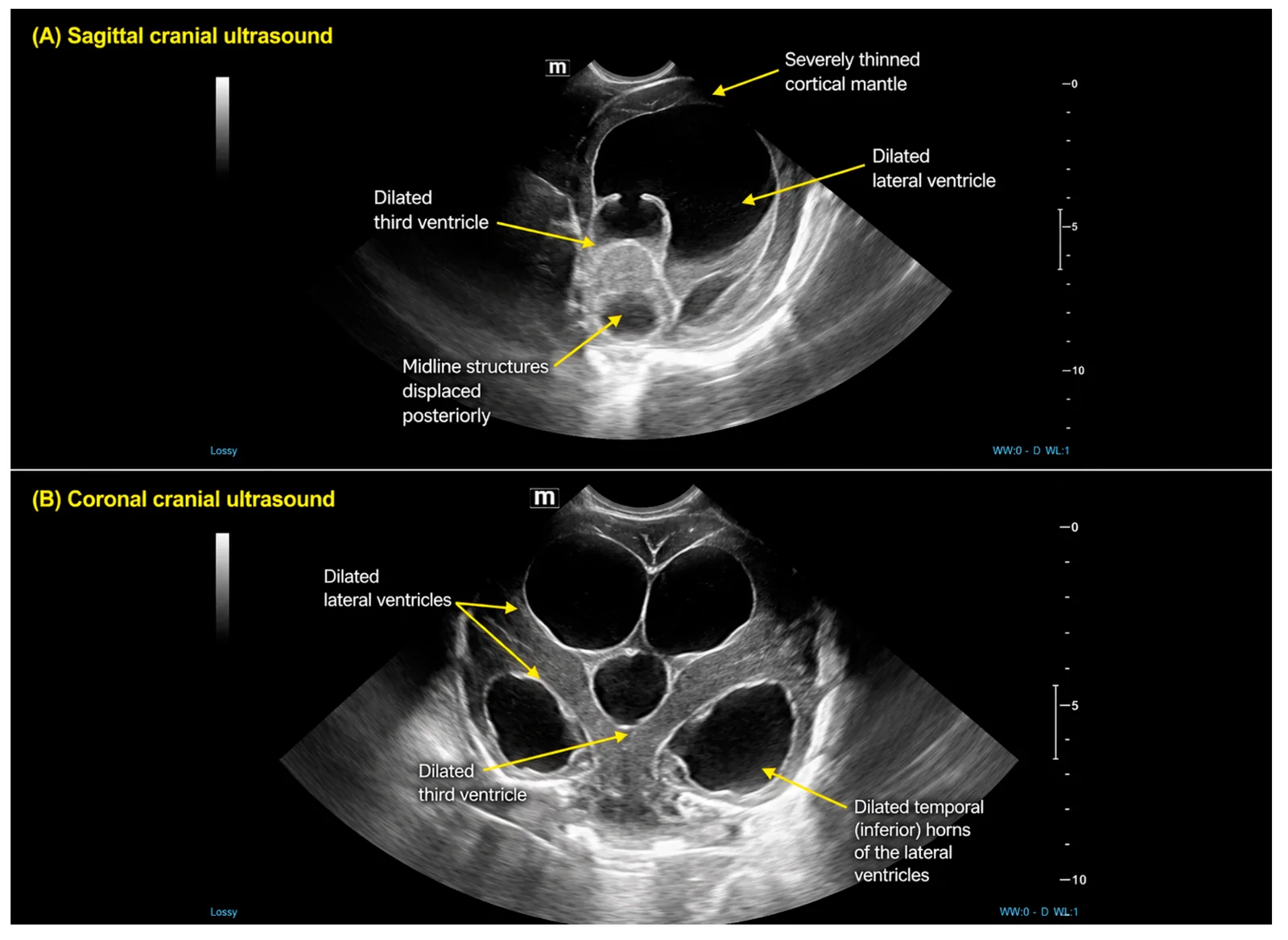
Representative cranial ultrasonography obtained on 26 August 2024, demonstrating severe post-hemorrhagic hydrocephalus with marked ventriculomegaly. (**A**) Sagittal image demonstrating severe enlargement of the ventricular system with marked cortical mantle thinning. (**B**) Coronal image demonstrating marked dilatation of both lateral ventricles and the third ventricle with distortion of normal ventricular anatomy.

Cranial ultrasonography performed on 26 August 2024 demonstrated severe post-hemorrhagic hydrocephalus characterized by marked ventriculomegaly. The sagittal view showed marked enlargement of the ventricular system with severe thinning of the cortical mantle. The coronal view confirmed marked dilatation of both lateral ventricles and the third ventricle, with distortion of the normal ventricular anatomy. Representative cranial ultrasound images are shown in Figure 2.

**Figure 3:**
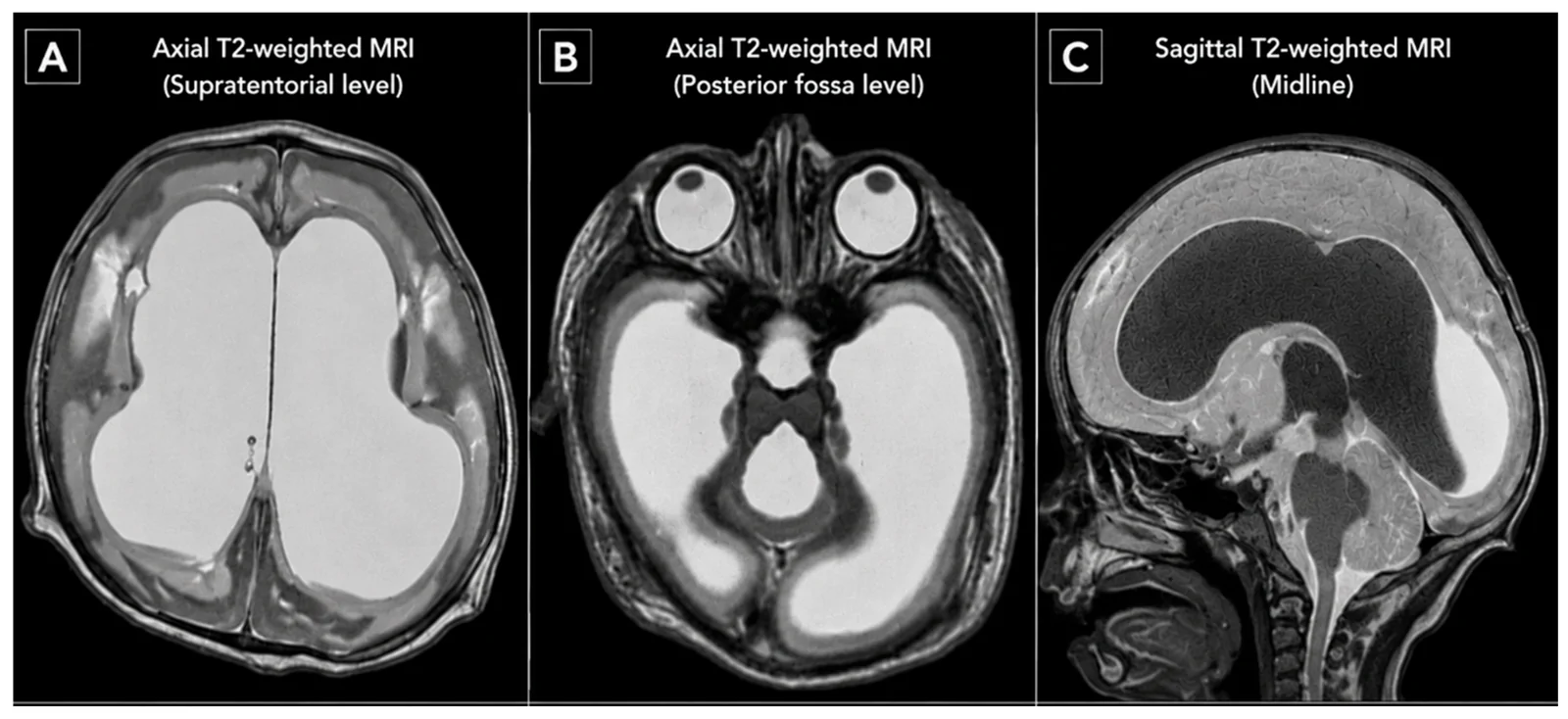
Representative baseline brain MRI obtained on 23 August 2024, at approximately 4 weeks of age demonstrating severe Grade IV germinal matrix hemorrhage with evolving post-hemorrhagic hydrocephalus. (**A**) Axial T2-weighted image showing marked dilatation of the lateral ventricles with early periventricular white matter injury. (**B**) Axial T2-weighted image at the posterior fossa level demonstrating ballooning of the fourth ventricle. (**C**) Midline sagittal T2-weighted image demonstrating severe communicating hydrocephalus with marked thinning of the corpus callosum.

Early brain MRI performed on 23 August 2024, at approximately 4 weeks of age, confirmed severe Grade IV germinal matrix hemorrhage with bilateral intraparenchymal extension and moderate-to-marked communicating hydrocephalus. The examination also demonstrated early periventricular cystic leukomalacia, marked thinning of the corpus callosum, ballooning of the fourth ventricle, and diffuse effacement of the cortical sulci without midline shift. These findings documented the severity of the patient’s underlying hemorrhagic brain injury and post-hemorrhagic hydrocephalus. Representative MRI images are shown in Figure 3.

**Figure 4:**
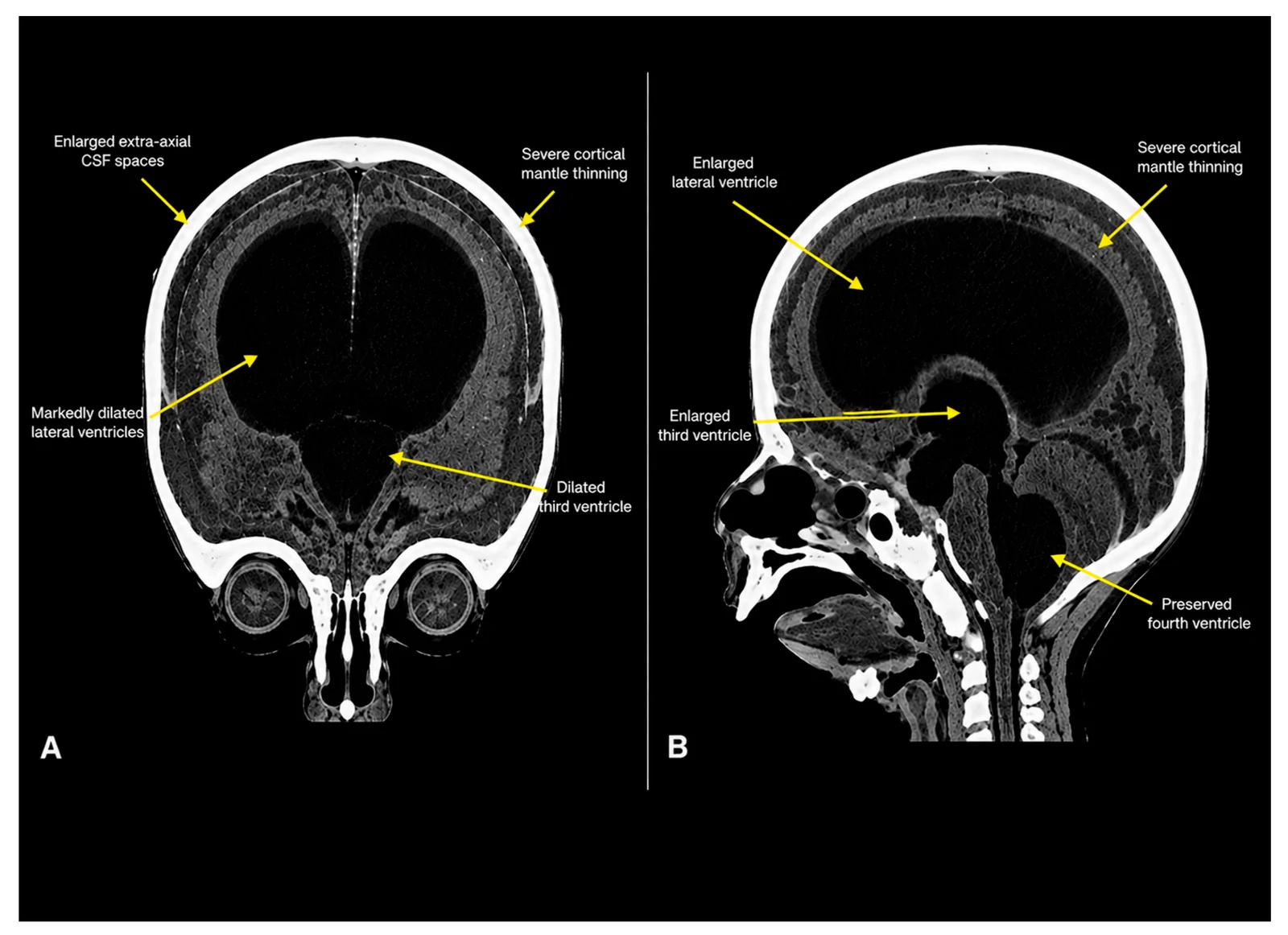
Historical cranial CT obtained on 19 August 2024 demonstrating severe hydrocephalus. (**A**) Coronal image demonstrating marked dilatation of the lateral and third ventricles, severe cortical mantle thinning, and enlarged extra-axial cerebrospinal fluid spaces. (**B**) Sagittal image demonstrating marked ventricular enlargement and severe cortical mantle thinning.

Historical cranial CT obtained on 19 August 2024 demonstrated severe hydrocephalus with marked dilatation of the lateral and third ventricles, profound cortical mantle thinning, and enlarged extra-axial cerebrospinal fluid spaces. Representative coronal and sagittal images from this examination are presented in Figure 4. During the current admission, CT examinations obtained on 27 February and 3 March 2026 were used for serial assessment; the latter demonstrated evolving bilateral extra-axial collections with features suggestive of a subacute subdural hemorrhage.

## 5. Diagnostic Considerations

The patient’s presentation prompted consideration of several potentially life-threatening causes of acute neurological deterioration. VP shunt-related infection remained an important diagnostic consideration because of the localized erythematous swelling over the shunt reservoir, persistent fever, elevated inflammatory markers, CSF pleocytosis with markedly elevated protein and CSF glucose of 2.0 mmol/L, and progressive neurological deterioration. However, these findings were nonspecific, and repeated blood, urine, wound, and shunt-derived CSF cultures remained negative. Accordingly, a VP shunt-related infection was not microbiologically confirmed. Culture-negative shunt infection, including infection associated with biofilm-forming organisms, remained a possibility but could not be established on the available findings [12,14,15].

Mechanical VP shunt malfunction was also considered because of recurrent vomiting, lethargy, irritability, and neurological deterioration. Initial CT demonstrated no significant interval increase in ventricular size, which argued against radiographically overt progressive hydrocephalus but did not independently exclude shunt dysfunction. Ventricular size may remain unchanged in some children with shunt malfunction, particularly in the setting of reduced ventricular compliance [9,10,11]. Subsequent neuroimaging demonstrated bilateral extra-axial fluid collections with features suggestive of a subacute subdural hemorrhage, providing an alternative intracranial explanation for the patient’s neurological deterioration. Given the patient’s history of repeated CSF diversion procedures and previous chronic subdural fluid collections, shunt over drainage was also considered as a potential mechanism contributing to the development or enlargement of the extra-axial collections. However, the available clinical and radiological data did not establish a causal relationship between shunt function and the collections, and over drainage could not be confirmed.

Several competing diagnoses were considered concurrently. Status epilepticus represented a potential contributor to the persistent alteration in consciousness and subsequent clinical deterioration; however, electroencephalography was not performed, limiting assessment for ongoing or non-convulsive seizure activity. Given the patient’s known secondary adrenal insufficiency, adrenal crisis or endocrine decompensation was also considered during the acute illness, prompting administration of stress-dose corticosteroids. Aspiration pneumonia following recurrent vomiting and prolonged seizures likely contributed to the respiratory deterioration and systemic inflammatory response. Collectively, the overlapping neurological, infectious, respiratory, endocrine, and possible shunt-related processes prevented definitive attribution of the acute deterioration to a single cause.

## 6. Management

Given the patient’s rapid neurological deterioration and the possibility of serious infectious, neurological, endocrine, and shunt-related causes, treatment was initiated immediately while diagnostic evaluation continued.

Following admission to the Pediatric Intensive Care Unit (PICU), management focused on neurological stabilization, empirical antimicrobial therapy, endocrine support, respiratory stabilization, and continuous neurosurgical assessment. Empirical broad-spectrum antimicrobial therapy with intravenous ceftriaxone, vancomycin, and clindamycin was administered while VP shunt-related infection, ventriculitis, meningitis, and other serious bacterial infections remained under consideration [12,13]. According to the available Infectious Diseases documentation, ceftriaxone was initiated on 27 February 2026 and clindamycin was administered from 3 to 6 March 2026. Vancomycin was also administered as part of the empirical antimicrobial regimen; however, its exact treatment dates could not be reliably established from the available documentation. Repeated blood, urine, wound, and CSF cultures remained negative, and no microbiological confirmation of VP shunt-related infection or ventriculitis was obtained.

Status epilepticus was managed aggressively with an intravenous levetiracetam loading dose followed by continuous midazolam infusion, while maintenance antiepileptic therapy was optimized under neurology supervision [16]. Given the patient’s known secondary adrenal insufficiency, stress-dose intravenous hydrocortisone was administered promptly during the acute illness and was subsequently tapered to maintenance replacement therapy following clinical stabilization [17].

Serial neurological examinations and repeat neuroimaging guided ongoing neurosurgical decision-making. Follow-up CT demonstrated bilateral extra-axial collections with imaging features suggestive of a subacute subdural hemorrhage. Emergency neurosurgical intervention was deferred because there was no radiological evidence of acute shunt displacement or life-threatening mass effect. Conservative management with close neurological observation was therefore continued while arrangements were made for further assessment at a tertiary pediatric neurosurgical center.

Aspiration pneumonia was managed with antimicrobial therapy and respiratory support. Episodes of systemic hypertension associated with intermittent bradycardia raised clinical concern for increased intracranial pressure and were managed with oral amlodipine. However, intracranial hypertension was not objectively confirmed by opening-pressure measurement.

Management remained directed toward multiple potential contributors to the acute deterioration rather than a definitively established VP shunt-related diagnosis.

## 7. Clinical Outcome

Following comprehensive multidisciplinary management in the Pediatric Intensive Care Unit (PICU), the patient’s clinical condition gradually improved. Neurological stabilization was achieved with seizure control, empirical antimicrobial therapy, stress-dose corticosteroid replacement, respiratory support, and close neurological monitoring.

After several days of mechanical ventilatory support, respiratory function progressively recovered, allowing successful extubation on 6 March 2026. He subsequently maintained satisfactory oxygenation on room air without further episodes of respiratory compromise.

Serial laboratory investigations demonstrated gradual normalization of inflammatory markers and improvement in systemic biochemical abnormalities. Despite repeated microbiological sampling throughout hospitalization, blood, cerebrospinal fluid, wound, and urine cultures remained negative, and a VP shunt-related infection was therefore not microbiologically confirmed. No definitive mechanical shunt obstruction or displacement was demonstrated on the available imaging. The principal evolving radiological abnormality was the development of bilateral extra-axial fluid collections with features suggestive of a subacute subdural hemorrhage. Accordingly, no single etiology of the acute neurological deterioration was definitively established before discharge and referral for tertiary neurosurgical evaluation, and possible shunt-related pathology, the evolving extra-axial collections, status epilepticus, systemic infection, and endocrine or metabolic decompensation remained competing or potentially overlapping contributors.

Neurologically, the patient returned to his pre-admission baseline. His Glasgow Coma Scale (GCS) improved progressively, with spontaneous eye opening, improved responsiveness to caregivers, and recovery of his usual level of interaction. No further episodes of status epilepticus occurred following stabilization, and maintenance antiepileptic therapy was continued under neurology supervision.

Repeat neuroimaging continued to demonstrate chronic hydrocephalus with persistent bilateral extra-axial fluid collections with features suggestive of a subacute subdural hemorrhage. Emergency neurosurgical intervention was not indicated because of the patient’s neurological improvement and stable clinical condition, although continued neurosurgical surveillance was required.

Given the complexity of his neurosurgical history, further evaluation at a tertiary pediatric neurosurgical center was recommended. At the time of discharge, the patient was hemodynamically stable and continued receiving maintenance hydrocortisone, antiepileptic therapy, baclofen for spasticity, amlodipine for blood pressure control, and enteral nutritional support. The family elected to proceed directly to the tertiary center for continued specialist assessment and management of his complex shunt-dependent hydrocephalus. Continued multidisciplinary follow-up involving pediatric neurosurgery, neurology, endocrinology, rehabilitation medicine, nutrition, and developmental services was recommended.

## 8. Discussion

Children with post-hemorrhagic hydrocephalus (PHH) who require ventriculoperitoneal (VP) shunts remain vulnerable to infection, mechanical failure, obstruction, over-drainage, and repeated surgical revision. Recognition of these complications is important because delayed diagnosis and treatment may result in substantial neurological morbidity. However, symptoms such as vomiting, lethargy, altered consciousness, fever, and seizures are nonspecific, particularly in children with severe pre-existing neurological impairment and multiple systemic comorbidities [1,2,3,4,5,6,7,8,9,10,11,12,13].

The principal feature of this case was the difficulty in determining the cause of acute neurological deterioration. VP shunt-related infection or malfunction was clinically considered because of the patient’s shunt dependence, localized erythematous swelling and tenderness over the reservoir, vomiting, altered consciousness, inflammatory abnormalities, and abnormal CSF findings. Nevertheless, neither shunt infection nor mechanical malfunction was definitively established. Repeated blood, CSF, wound, and urine cultures remained negative, and the available microbiological investigations did not confirm infection, while initial CT demonstrated no significant interval increase in ventricular size. These findings did not independently exclude shunt-related pathology, but they substantially limited diagnostic certainty and required consideration of competing explanations [9,10,11,12,13,14,15].

Interpretation of the CSF findings was similarly challenging. The patient had lymphocytic pleocytosis, markedly elevated CSF protein, and CSF glucose of 2.0 mmol/L; however, these abnormalities were not specific for acute bacterial shunt infection, particularly given his history of recurrent neonatal pyogenic meningitis and chronically elevated baseline CSF protein. Although culture-negative healthcare-associated ventriculitis and shunt infections have been described, including in association with prior antimicrobial exposure, low organism burden, and biofilm formation, negative cultures should not themselves be interpreted as evidence of infection. Rather, microbiological results must be considered together with the clinical presentation, CSF profile, imaging, and treatment history [12,13,14,15,18,19].

Neuroimaging added further uncertainty. Initial CT demonstrated no significant interval change in ventricular size despite neurological deterioration. Shunt malfunction without ventricular enlargement has been reported in children with chronic hydrocephalus, and ventricular size alone therefore cannot reliably exclude dysfunction [9,10,11]. However, unchanged ventricular size also did not establish occult shunt malfunction in this patient. Subsequent imaging demonstrated bilateral extra-axial fluid collections with features suggestive of a subacute subdural hemorrhage, providing an alternative intracranial process that may have contributed to the neurological presentation. Shunt over drainage is a recognized mechanism for the development of subdural or other extra-axial fluid collections in shunted patients [20] and warranted consideration in this case, particularly given the patient’s history of multiple CSF-diversion procedures and previous chronic subdural collections. However, the available imaging and clinical data were insufficient to demonstrate that the evolving collections resulted from over drainage, and this mechanism therefore remained a differential consideration rather than an established diagnosis. Clinical concern for increased intracranial pressure arose during the admission; however, intracranial hypertension was not objectively confirmed by opening-pressure measurement. Fundoscopic examination was attempted, but the fundus could not be adequately visualized because of reactive pupils; therefore, papilledema could neither be confirmed nor excluded. These limitations precluded objective confirmation of raised intracranial pressure.

Importantly, several non-shunt-related processes could have contributed to the acute deterioration. Status epilepticus itself may cause prolonged impairment of consciousness and systemic physiological disturbance, particularly in a child with severe structural brain injury and pre-existing epilepsy [16]. Electroencephalography was not performed during the reported episode; therefore, ongoing or non-convulsive seizure activity could not be objectively excluded as a contributor to persistent altered consciousness. Secondary adrenal insufficiency created an additional potential source of physiological decompensation during acute illness and warranted stress-dose corticosteroid therapy [17]. Aspiration pneumonia following recurrent vomiting and prolonged seizures likely contributed to respiratory failure and systemic inflammation. The evolving extra-axial collections provided an additional structural intracranial consideration. Consequently, the available evidence does not permit the acute neurological deterioration to be attributed confidently to a single mechanism. Among the objective findings, the evolving bilateral extra-axial collections represented the principal interval radiological abnormality during the acute course. However, their causal contribution to the neurological deterioration, including whether they were related to shunt over drainage, could not be established from the available data. Thus, the final clinical interpretation was multifactorial acute neurological deterioration of uncertain primary etiology rather than confirmed VP shunt infection or mechanical shunt failure.

Management therefore addressed several potentially serious processes simultaneously. Empirical antimicrobial therapy was administered while VP shunt-related infection, ventriculitis, meningitis, and other serious bacterial infections remained under consideration [12,13,15]. Repeated microbiological investigations remained negative, and no microbiological confirmation of VP shunt-related infection or ventriculitis was obtained. Status epilepticus was treated aggressively, stress-dose corticosteroids were administered because of known adrenal insufficiency, respiratory support was provided, and serial neurological and neurosurgical assessments were performed [16,17]. The patient’s subsequent improvement following these concurrent interventions cannot establish which process was primarily responsible for the deterioration. Rather, the clinical course illustrates the importance of stabilizing critically ill children while diagnostic uncertainty remains unresolved.

This case therefore emphasizes a broader diagnostic principle rather than establishing a specific shunt complication. The limitations of microbiological cultures in suspected shunt infection and the occurrence of shunt malfunction without ventricular enlargement are already recognized in the literature [9,10,11,12,13,14,15]; accordingly, these observations are not presented as novel findings of this report. Rather, the educational value of this case lies in illustrating the diagnostic difficulty created by several overlapping neurological and systemic processes in a severely neurologically impaired, shunt-dependent child whose acute deterioration could not be attributed to a single established cause. In this setting, negative cultures and unchanged ventricular size should not be used in isolation either to exclude or to establish shunt-related pathology. Evaluation should remain broad and include infectious, mechanical, seizure-related, structural, respiratory, endocrine, and metabolic causes. Serial neurological assessment, appropriate laboratory investigation, repeat neuroimaging, multidisciplinary evaluation, and timely treatment of immediately threatening conditions are particularly important when no single investigation provides a definitive explanation [9,10,11,12,13,14,15,16,17,18,19,20,21,22].

## 9. Patient Perspective

The infant’s parents described the prolonged hospital course and the need for repeated neurosurgical interventions as a significant source of anxiety. They expressed appreciation for the regular communication provided by the multidisciplinary team and understood the need for continued long-term follow-up because of the possibility of future shunt-related complications and neurodevelopmental concerns.

## 10. Conclusions

This case highlights the diagnostic complexity of acute neurological deterioration in a child with shunt-dependent post-hemorrhagic hydrocephalus and multiple neurological and systemic comorbidities. No single etiology was definitively established before discharge and referral for tertiary neurosurgical evaluation. VP shunt-related infection and mechanical malfunction remained diagnostic considerations but were not confirmed, while evolving bilateral extra-axial collections, status epilepticus, respiratory infection, and endocrine or metabolic decompensation represented competing or potentially overlapping contributors. The final clinical interpretation was therefore multifactorial acute neurological deterioration of uncertain primary etiology rather than a confirmed VP shunt complication.

Negative microbiological cultures and unchanged ventricular size on initial neuroimaging should not be interpreted in isolation as either excluding or establishing VP shunt-related pathology. Clinical evaluation should integrate serial neurological assessment, laboratory findings, repeat neuroimaging, consideration of alternative diagnoses, and multidisciplinary assessment. When the underlying cause remains uncertain, management should prioritize stabilization and timely treatment of potentially life-threatening conditions while diagnostic evaluation continues.

## Figures and Tables

**Table 1:** Laboratory and CSF findings during the acute presentation.

Diagnostic Parameter	Observed Value	Clinical Interpretation
CSF Protein	>2500 mg/L	Markedly elevated
CSF Glucose	2.0 mmol/L	CSF glucose 2.0 mmol/L; paired serum glucose unavailable
CSF White Blood Cells	49 cells/mm^3^ (99% lymphocytes)	Lymphocytic pleocytosis
Serum CRP	23.7 → 30.4 mg/L	Progressive systemic inflammatory response
White Blood Cell Count	15.75 × 10^3^/μL	Leukocytosis with neutrophilia
AST/ALT	194 U/L/95 U/L	Mild transaminitis consistent with systemic illness

## Data Availability

The data presented in this study are available on reasonable request from the corresponding authors. The data are not publicly available due to patient privacy and ethical restrictions.
